# Models of Language and Multiword Expressions

**DOI:** 10.3389/frai.2022.781962

**Published:** 2022-02-17

**Authors:** Pablo Contreras Kallens, Morten H. Christiansen

**Affiliations:** ^1^Department of Psychology, Cornell University, Ithaca, NY, United States; ^2^Interacting Minds Centre and School of Communication and Culture, Aarhus University, Aarhus, Denmark; ^3^Haskins Laboratories, New Haven, CT, United States

**Keywords:** multiword chunks, Construction Grammar, grammar, language acquisition, language processing, lexical frames, multiunit expressions

## Abstract

Traditional accounts of language postulate two basic components: words stored in a lexicon, and rules that govern how they can be combined into meaningful sentences, a grammar. But, although this words-and-rules framework has proven itself to be useful in natural language processing and cognitive science, it has also shown important shortcomings when faced with actual language use. In this article, we review evidence from language acquisition, sentence processing, and computational modeling that shows how multiword expressions such as idioms, collocations, and other meaningful and common units that comprise more than one word play a key role in the organization of our linguistic knowledge. Importantly, multiword expressions straddle the line between lexicon and grammar, calling into question how useful this distinction is as a foundation for our understanding of language. Nonetheless, finding a replacement for the foundational role the words-and-rules approach has played in our theories is not straightforward. Thus, the second part of our article reviews and synthesizes the diverse approaches that have attempted to account for the central role of multiword expressions in language representation, acquisition, and processing.

## Introduction

Each of us in our lifetime will only ever hear or speak a finite number of sentences, yet we can understand and produce an infinite number of sentences as long as they are (reasonably) grammatical and we know the words that appear in them. As already noted nearly 200 years ago, this celebrated aspect of human language requires us to “make infinite employment of finite means” (Von Humboldt, 1999, p. 91). But how do we do this? What are the finite means that underly our infinite capacity for language?

The classic solution to these questions is to postulate a bipartite distinction in the structure of language: a *lexicon* and a *grammar* (Chomsky, 1965), where the former stores the words of a language and the latter specifies how they can be combined. Thus, not unlike how a limited number of types of atoms can combine into the unlimited infinitude of the universe, a limited number of words can be combined into an unlimited set of sentences. And, just like how the laws of physics govern these combinations such that not *any* molecule is possible, the grammar specifies the rules that govern which sentences are and are not possible. This sets up a fundamental difference between *accessing* an individual item and *generating* a combination of them.

Although intuitive and powerful, this words-and-rules perspective (Pinker, 1999) does not account for actual language use, even though it has been proposed as such (e.g., Pinker, 1994; Levelt, 1998; Ullman, 2001)^1^. A vast proportion of the sentences uttered by speakers appears to be built not from isolated words or morphemes put together on the fly but from stored and relatively fixed multiunit sequences that span several of them (e.g., Jackendoff, 1997; De Cock et al., 1998). In this review, we argue that the ubiquity of such multiunit sequences requires a reevaluation of how key components of language are conceived, with important implications for theories of acquisition, processing, and representation.

The article has two main parts. The first one reviews the mounting evidence of the ubiquitous role of sequences spanning multiple lexical and/or morphemic units in language use. We start by reviewing the research about the processing of idiomatic and formulaic expressions, and how it challenges the distinction between lexicon and grammar. Then, we argue that, in light of more recent work, these two types of expressions are not so special: familiarity effects with multiword sequences can be found across all language. In the second half, we bite the bullet of what this research suggests and review alternative accounts of language that do not assume a distinction between lexicon and grammar at any level. For expository purposes, we divide this presentation between acquisition and processing. With this, we aim to sketch a conception of language more deeply rooted in the patterns of actual language use.

## The Ubiquity of Multiunit Expressions

### Idiomatic and Formulaic Expressions

Historically, the first encounter of the traditional words-and-rules approach with units spanning more than one lexical item was in the research on idiomatic expressions. In the traditional definition, an idiom is a phrase whose meaning is not a function of its components (e.g., Fraser, 1970; Weinreich, 1980; Pinker, 1999), that is, it is not compositional. For example, there is nothing in the individual words nor the syntactic combination of “he,” “bit,” “the,” and “bullet” that could suggest that the sentence “he bit the bullet” means that someone accepted painful consequences (and not that the person actually bit a bullet).

This poses a problem for the traditional words-and-rules approach because understanding or producing a sentence involves parsing the syntactic structure to determine the relationships between lexical items (Chomsky, 1970). But an idiom's semantic and syntactic structure are not straightforwardly related to its meaning. Thus, using grammatical composition of individually stored items does not have the same appeal for “bite the bullet” as it could have for the structurally and semantically identical “ride the bike” and “accept the harsh consequences” respectively. The same difficulty is expressed in the “syntactic frozenness” of some idiomatic expressions: idioms vary in how well their meaning is preserved after purely syntactic transformation. In the classic example, “kick the bucket” cannot be used in passive form, as “the bucket was kicked” does not have the same meaning of “to die” (Katz and Postal, 1963). In contrast, the idiom “make up your mind” can be passivized, as in “your mind can be made only by you” (Fraser, 1970).

The words-and-rules approach traditionally dealt with this anomalous behavior (Chafe, 1968) wherein idioms straddle the distinction between accessed and generated pieces of language by pushing them into the lexicon. This modified conception views the lexicon not as a repository of *words* but of any accessed item—anything whose meaning has to be memorized due to being either atomic (morphemes such as “-ed”), arbitrary (words), or non-compositional (idioms) (Pinker, 1999). In this sense, the lexicon becomes a “prison” (Di Sciullo and Williams, 1987) for elements that do not conform to or cannot be generated by the grammar (see also Chomsky, 1995; Jackendoff, 1997) – a container of *listemes* as opposed to individual words (Di Sciullo and Williams, 1987).

This approach found some initial empirical support in psychology—mainly in findings that idioms are processed faster than their literal counterparts (Swinney and Cutler, 1979; Gibbs, 1980; Tabossi et al., 2009; see Nunberg et al., 1994; for an approach from linguistics). However, recent work has shown that controlling for substring frequency and length eliminates the advantage of idioms over meaningful compositional phrases (Jolsvai et al., 2020). Moreover, eliminating any role for the components of idioms in their processing is inconsistent with empirical evidence. Firstly, some idiomatic expressions are more frozen than others, which makes it difficult to assume that they are all stored and accessed as wholes in the same manner (Mel'cuk, 1995; Van de Voort and Vonk, 1995). In fact, syntactically frozen idioms such as “kick the bucket” are not completely frozen, as some variations (e.g., “kicking the bucket”) are still allowed^2^. Secondly, “compositionality” is a second continuum in addition to “frozenness,” as some idiomatic expressions can be seen as compositions of metaphorical elements (Gibbs, 1980; Geeraerts, 1995) and how compositional an idiom is has been shown to affect how it is processed (Gibbs et al., 1989; Gibbs, 1995). Finally, the elements of an idiom are processed sequentially, and they can play a role in their recognition. The idiomatic meaning of a sentence is triggered when a disambiguating key word is recognized (Tabossi et al., 2009; Cacciari, 2014). Therefore, the claim that idioms are memorized as wholes and stored in the lexicon—thus maintaining the plausibility of the distinction between lexicon and grammar—is untenable.

Idiomatic expressions thus threaten the distinction between lexicon and grammar because they exhibit behavior traditionally ascribed to both parts of the divide. But it could be argued that they are a relatively rare linguistic exception—that is, that they belong in the “periphery” of language as opposed to the “core” (Chomsky, 1995; see Culicover, 1999; for a criticism of this distinction). However, idioms are only a small subset of a much more numerous kind of multiunit sequence: formulaic expressions. These are relatively fixed expressions commonly used to communicate specific meanings in a proportion overwhelmingly higher than other grammatical alternatives (Wray, 2002). Idioms are a particularly non-compositional and syntactically frozen subset of formulaic expressions (Wray and Perkins, 2000; Conklin and Schmitt, 2012).

Learning the formulaic expressions of a particular language—conventionally used to express certain meanings – is a key step in becoming a proficient language user (Pawley and Syder, 1983). As an intuitive example, consider the difference between the two expressions: “my grandma's sick” and “the mother of my father is stricken by disease.” Even though both roughly mean the same thing, preferential use of the former over the latter is a key feature of native-like language use (Pawley and Syder, 1983). Apart from idioms, formulaic expressions also include “lexical bundles” (“in the middle of the,” Tremblay et al., 2011), complex propositions and verbs (“in support of” and “take up,” respectively, Siyanova-Chanturia, 2013), turns of phrase (“a priori,” “for whatever reason,” Mel'cuk, 1995), collocations and binomials (“black coffee” and “bride and groom,” respectively, Siyanova-Chanturia, 2013), full phrases (“how can I ever repay you?” Wray and Perkins, 2000), and even longer sequences of linguistic material such as songs or poems.

Formulaic expressions have been found to constitute a considerable portion of the language use of native speakers. When attempting to count them, various researchers have estimated the number of fixed phrasal expressions in the tens of thousands (Weinreich, 1980; Jackendoff, 1997), with multiword entries making up more than 40% of WordNet's entries (Sag et al., 2002). Estimates of formulaic expressions in corpora have returned various surprisingly high estimates. Van Lancker Sidtis and Rallon (2004) found that roughly a quarter of the expressions in the dialogue of a film could be categorized as either idiomatic or formulaic. Erman and Warren (2000) define formulaicity in the context of lexical choice: slots in sentences constrained by their occurrence in fixed expressions are defined as “formulaic” choices (Sinclair, 1991). They found that more than half of the slots in the extracts they selected are filled with formulaic expressions. This pattern can even be found in heavily agglutinating languages such as Turkish (Durrant, 2013), where a high proportion of morphemes co-occur in predictable, formulaic patterns instead of the nigh-infinite number of possible combinations.

More quantitative approaches are based on the predictability of the elements of a sequence (e.g., Columbus, 2010; Church, 2013; Kumova Metin, 2018). Intuitively, the elements of a formulaic sequence are more predictable given the previous elements (“bucket” given “kick the”) than the elements of a novel sentence (“mom” given “call your”). This predictability can be operationalized as the mutual information between the words of a phrase (Church and Hanks, 1989): a higher mutual information score between two words suggests higher predictability. Based on this notion, Nelson (2018) analyzed several corpora and compared the mutual information between the components of all occurring bigrams and their expected baseline based on frequency. His estimates confirm the ranges mentioned above, with a high 50% to a low of 20% for the proportion of bigrams that can be considered formulaic.

Formulaic expressions are also key to enabling the fluency that characterizes native-like use of language (Pawley and Syder, 1983). Considering the high processing demands that language use imposes on speakers and hearers (Christiansen and Chater, 2016), unconstrained choice of lexical items and from-scratch sentence production is unlikely to result in the flow of speech associated with native proficiency. Formulaic expressions allow speakers to achieve this fluency by limiting the choices about what phrases to use when expressing particular meanings, what words to use in them, and in what order to use them (Sinclair, 1991). Consistent with this, Wood (2006) found that the use of formulaic sequences helped second-language learners of English to attain fluency by minimizing the number of pauses when retelling stories. Other research has also emphasized this link between fluency and formulaicity: for example, Kuiper (2004) argues that much of the language in fluent contexts like oral poetry, auctions, and ritualized interactions relies on combining pre-generated phrases that minimize the processing load associated with creating novel phrases.

Similar to idioms, formulaic expressions exhibit anomalous behavior with respect to the base distinction between storage and generation that underlies the word-and-rules perspective. They can be continuous or discontinuous (Siyanova-Chanturia and Pellicer-Sanchez, 2018), lying thus in a continuum of fixedness (e.g., “what is/are X/you up to?”; see Wray and Perkins, 2000, for more examples). They can also be non-compositional: what is being cleaned by whom is different in the structurally identical collocations “carpet sweeping” (the carpet by a brush) and “vacuum cleaning” (something by a vacuum) (Cacciari, 2014). Thus, they can also be placed in a continuum of compositionality. However, in contrast to idiomatic expressions, they are used frequently and constitute a large part not only of actual language use but of what it means to learn it. This further undermines the solution of storing idioms in the lexicon to defend the divide between it and the grammar and suggests that the problem with this account is deeper than what can be comfortably exiled to the periphery of language.

However, it could be argued that formulaic expressions are a purely linguistic phenomenon—that is, artifacts present in descriptions of language use that have no bearing on cognitive machinery. Thus, to complete the point being made on the plausibility of the distinction between lexicon and grammar, the pervasiveness of formulaic expressions must be shown to have some psycholinguistic counterpart.

The Declarative/Procedural account (Ullman, 2001)—a cognitive and neural implementation of the words-and-rules perspective—argues that a distinction between the lexicon (declarative) and the grammar (procedural) can be made based on frequency effects. If the frequency of an item affects how it is processed, language users must have some stored representation to which the effect can be associated. A related position sees frequency effects as reflecting “entrenchment” (Divjak and Caldwell-Harris, 2015), where high frequency suggests more opportunities to become familiar with an item, thus consolidating the memories of them into their own representations. In other words, frequency effects strongly suggest that an item is accessed. Therefore, in a words-and-rules account, they should not be observed for phrases that could be generated by rule-following processes regardless of their frequency of occurrence.

This, however, is in stark contradiction to much recent empirical work showing a processing advantage for formulaic language. In this case, formulaic sequences are operationalized as a combination of words or morphemes that co-occur together more often than would be expected by chance (Church, 2013; Constant et al., 2017) and with a high enough absolute frequency as a unit to assume familiarity with it (Wray, 2012). Several reviews (Conklin and Schmitt, 2012; Siyanova-Chanturia, 2013; Constant et al., 2017) document this advantage: a consistent observation that they are comprehended significantly faster than matched low-frequency sentences. Tremblay et al. (2011) found, for example, that lexical bundles such as “in the middle of the” were read faster and recalled with higher accuracy than sequences matched for length such as “in the front of the” during self-paced reading experiments. This advantage is also observed in an agglutinative language: Lõo et al. (2018) found that high-frequency “complete” forms in Estonian (as opposed to high-frequency uninflected lemmas) have shorter reaction times in a lexical decision task.

The advantage in processing is also found in production. Bannard and Matthews (2008) found that children's accuracy in recalling and the duration of pronunciation of high frequency (“sit in your chair”) expressions were higher and faster than low-frequency controls with a different final word (“sit in your truck,” Bannard and Matthews, 2008). Arnon and Cohen Priva (2013) found a similar production advantage for highly frequent phrases in adults. These results are related to the phenomenon of phonological reduction, in which the duration of individual words during sequences in which they occur frequently is reduced diachronically (Gahl et al., 2012). Bybee and Scheibman (1999) document this in the sequence “don't,” which during conversation is reduced in the most frequent uses such as “I don't know” into “I dunno.”

Eye-tracking provides further evidence for this advantage. Underwood et al. (2004) found that formulaic sequences have shorter and less frequent fixations on individual words than non-formulaic expressions. This effect was replicated in English binominals (Siyanova-Chanturia et al., 2011): literal and idiomatic high-frequency binomials were read faster and with fewer fixations than novel controls by both L1 and L2 speakers. Cutter et al. (2014) showed that the fixation times on the final word of a collocation are shorter when both words are available for preview, suggesting that the recognition of the first word facilitates the processing of the whole collocation. Similarly, for Chinese collocations (Jiang et al., 2020) the reading time of both the last word and whole phrase was lower for phrases that included high-frequency collocations.

Observations of these effects also underline the continuity between idioms and formulaic expressions. Columbus (2010) found no sharp distinction in reading nor fixation times between idiomatic and non-idiomatic formulaic expressions, even though both present an advantage in comparison to novel literal expressions. Carrol and Conklin (2020) also found that the reading time advantages of binomials and collocations in comparison to controls are similar, although not identical, to that of idiomatic expressions. Finally, Jolsvai et al. (2020) found that idioms and non-idiomatic three-word sequences—carefully controlled for frequency and meaningfulness—were processed at the same speed, both being faster than phrasal fragments.

### The Pervasiveness of Familiarity

A prominent proposal to integrate formulaic expressions into an account of language posits two different and separate language systems, the dual-systems approach (Sinclair, 1991; Wray, 2002; Van Lancker Sidtis, 2012b). One of these systems follows the words-and-rules approach: the grammar generates syntactic structures with slots that can be filled by accessing the memorized individual elements in the lexicon. This system describes how people creatively generate novel sentences and is impervious to frequency effects. By contrast, the second system consists of a large repository of stored high-frequency sequences, or formulas, that are used “holistically” (Wray and Perkins, 2000) even if they might look, on the surface, to be the product of a generative process.

Frequency effects are explained by this proposal because, if formulaic sequences are accessed as unanalyzed wholes, their meaning would be understood more quickly than if they had to be generated. This “economy of processing” (Perkins, 1999; Kuiper, 2004; Conklin and Schmitt, 2012) justifies the existence of a second system dealing with familiar phrases in contexts where speed of processing and fluency, among others, are necessary or beneficial (see e.g., Pawley and Syder, 1983, p. 49; Wray, 2002, p. 105). The advantage being, of course, that this can be achieved without sacrificing the core appeal of the words-and-rules perspective in explaining the creative use of novel language.

The proponents of the dual-systems approach have presented additional evidence for this qualitative division of processing labor. For example, formulaic sequences are perceived to be more “phonologically coherent” (Hickey, 1993) than novel sequences, presenting fewer pauses between the elements as they are accessed as a whole (see Lin, 2010 for a review). The last word of formulaic sequences has been argued to elicit a reliably smaller N400 component in EEG studies (Siyanova-Chanturia, 2013), suggesting a reduced cognitive load (Kutas and Federmeier, 2011). Finally, they have pointed to an alleged lateralization, with the formulaic system relying on the right hemisphere and basal ganglia, and the one for novel sequences on the left hemisphere (Van Lancker Sidtis, 2012a). This evidence could suggest that formulaic expressions somehow have a special status in language processing, separate from the generation of regular sentences.

However, this further distinction does not hold under further scrutiny. First, it presents various conceptual challenges. As it has been recognized even by the proponents (and former proponents) of the dual-systems approach (Myles et al., 1998; Conklin and Schmitt, 2012; Wray, 2012), there is no observable difference between items produced by either of the systems. Formulaic sequences can be continuous or discontinuous, of any length, any frequency, and any degree of compositionality (e.g., see Siyanova-Chanturia and Pellicer-Sanchez, 2018). Therefore, any of the two systems could, at least in principle, be the sole substrate of language use. But from an evolutionary standpoint, fluency and rapidity of processing are the norm. Turn-taking in dialogic interaction is rapid and demanding (Levinson, 2016), and listeners need to deal expediently with the rapid torrent of linguistic input before its obliterated by new incoming information (Christiansen and Chater, 2016). Thus, it is difficult to assess how an extra system that does not adequately respond to those pressures could emerge, especially when there is already another system that can produce the same set of sequences. The burden of proof, then, passes from justifying the need for a formulaic system to the need for a lexicon and grammar.

Secondly, and more importantly given its consequences for the words-and-rules account, recent research has found no sharp distinction in the processing of high-frequency and low-frequency sequences. This is key for maintaining the divide between the two systems, thereby limiting the reach of the ubiquity of multiunit expressions. The complete argument to explain the processing advantage is thus: frequency facilitates processing beyond the reliably established domain of individual lexical items (Brysbaert et al., 2018) and extends to sequences of longer length, which suggests that they are learned and processed similarly to words. Furthermore, this effect is presumably a consequence of familiarity, with corpus frequency serving as its proxy (Divjak, 2019). A harsh boundary between accessed and generated language, then, necessitates a frequency threshold for the advantage (Arnon and Snider, 2010). Instead, what has been found is a continuum in which more frequent sequences generally have advantages over less frequent ones (Wray, 2012; Divjak and Caldwell-Harris, 2015).

In comprehension, Arnon and Snider (2010) found that the reaction times in a phrasal decision task using four-word sequences are lower when the frequency of the whole phrase is higher; this extends to a difference between middle- and low-frequency items. Furthermore, a model that included a continuous measure of frequency was a significantly better fit to their data than one that included a binary one. Caldwell-Harris et al. (2012) report a similar result in a perceptual identification task: the probability of identification was higher for higher frequency word pairs across the entire frequency spectrum, including an advantage of low-frequency legal pairs over very low frequency and random pairs. Jacobs et al. (2017) observe continuous facilitation of accuracy associated with phrase frequency in a free recall task. In production, Janssen and Barber (2012) find that the latency of production in a picture-naming task was continuously reduced depending on the frequency of the names of the targets. Thus, behaviorally, the evidence favors a continuum of familiarity across all sequences instead of a sharp division between high- and low-familiarity ones.

These behavioral conclusions have been further supported by ERP and eye-tracking studies that have studied more dense frequency ranges. Tremblay and Baayen (2010) show that the frequency of a four-word sequence continuously modulates early N1a and P1 components usually associated with frequency effects. A later study found that phrase frequency has a near-linear effect on the components' voltages (Hendrix et al., 2017). Similarly, Yi et al. (2017) suggest that the differences in reading and fixation time they report are not limited to high-frequency multiword sequences.

Moreover, the elements of multiunit sequences and the relationships between them play a significant role in the processing advantage, further underlining their continuity with idioms and undermining any notion that they are stored holistically. Indeed, Ellis et al. (2008) found an effect of mutual information above phrase frequency in the reaction times of native speakers in a phrasal decision task; this suggests that the processing advantage is mediated by the individual components of the sequence. In a more direct test, Arnon and Priva (2014) found that production is influenced by both word and phrase frequency separately. Even more, they interact: the effect of individual word frequency is lower for higher frequency phrases, and it did not disappear even in the highest phrase frequency quartile. Similarly, Tremblay and Tucker (2011) found that the variance in latency of onset in production of four-word sequences is explained by all four levels of n-gram frequency to different but considerable degrees.

There is also evidence of interaction between features that should belong exclusively in either of the systems in a two-systems approach. For example, idiomatic expressions can prime and be primed by their component words to a similar degree to non-idiomatic expressions (Sprenger et al., 2006). Furthermore, they can syntactically prime other sentences, both in particle placement (“pull off a robbery” priming “pull off my sweatshirt,” Konopka and Bock, 2009) and in argument structure (double object vs. prepositional datives, Snider and Arnon, 2012). These priming effects are important because idioms are at the far end of the formulaicity spectrum, and, as such, their belonging to a hypothetical holistic processing system should be uncontroversial; thus, if they show signals of having internal structure, it is implausible to argue that non-idiomatic formulaic expressions do not.

In the inverse direction of influence, frequency has been shown to affect processes previously attributed exclusively to rule-based generative processing. For instance, ordinarily, object relative clauses with embedded noun-verb combinations are harder to process than subject relative clauses (Gibson, 1998); however, when object relative clauses involve a personal pronoun, the difference is reversed. Reali and Christiansen (2007b) found that this pattern reflects the higher frequency of object relative clauses containing personal pronouns over their subject relative counterparts. Furthermore, the effect is modulated by the frequency of the specific combination of pronoun-verb that is embedded in the clause (e.g., “the detective who the attorney who [*I met* distrusted/*I distrusted* met] sent a letter on Monday night,” Reali and Christiansen, 2007a). Therefore, not even syntactic patterns are excluded from the phenomena that should only characterize a storage-based system.

In summary, evidence shows that the effects of familiarity with multiunit sequences cannot be isolated from the rest of language. Aside from the difficulty of identifying which sentences were produced using rules and which were not, the effects are pervasive across the entirety of language and include phenomena that would be considered purely syntactic. Thus, the ubiquity of effects related to multiunit expressions found at every length of sequence from bigrams to sentences, and across the whole spectrum of frequency, make the words-and-rules proposal wholly inadequate as a theory of actual language use. Communication seems to exist at the boundary (Wray, 2009) where no useful distinction can be drawn between formulaic and novel nor, more importantly, between lexicon and grammar.

The demise of a theory, however, is not enough. The phenomena presented up to this point are still in need of an explanation that can, at least in principle, also explain the phenomena identified by the words-and-rules perspective. Importantly, this alternative must rely on a single “system” that processes sequences across the complete spectrum of frequency, formulaicity, and idiomaticity, among others. In the remainder of this article, we outline an approach that aims to explain language use and describe language knowledge while eschewing the distinction between lexicon and grammar.

## Single-System Accounts of Language

### Acquisition

Accounts of language acquisition based on the words-and-rules proposal posit two separate developmental trajectories: one for the lexicon where sequences, mainly words, are memorized, and one for the grammar, where the capacity for syntactic and morphological generation of sequences is tuned to the language of the speaker (e.g., Guasti, 2002). A vast majority of these approaches also assume that a large proportion of this trajectory consists of the maturation of innate structures (Crain, 1991; Pinker, 1994). Lexicon and grammar characterize different phases of early language development, with a focus on the lexicon during the single-word phase and on the grammar during the subsequent multi-word utterance phase (Locke, 1997). A large challenge for children is to learn the connection between both components, such as assigning lexical items to a syntactic category like “noun” or “verb” (Bloom, 2000). Thus, acquiring productivity is divided into learning the elements to be combined, the rules for combining them, and the interfaces that allow for them to be used in tandem.

Multiunit expressions lie at the core of the alternative to this perspective. Consider first formulaic language: how much of what is seen as the maturation of combinatorial skills can be attributed to learning to the manipulation of fixed sequences? Peters (1977) documents the use of “proto-sentences” by a child with no evidence of analytic combinatory mechanisms. She deemed this to be a “Gestalt” strategy, situated in one extreme of a continuum of reliance on formulaicity, with “analytic” strategies on the opposite end. Relatedly, Clark (1974, 1977) proposed that part of children's ability to produce longer sentences can be attributed to their capacity to extract continuous parts of sequences and couple them with other ones. To achieve this, children have been shown to rely in part on the transitional probabilities between syllables, with low probability transitions indicating the presence of a word boundary (Saffran and Aslin, 1996). Information about the statistics of phonological regularities is then integrated with a plethora of other probabilistic cues (Christiansen et al., 2005). The segmented sequences are paired with information about the frequency of co-occurring elements, such that high-frequency sequences are segmented from low-frequency ones (Marcovitch and Lewkowicz, 2009; Saffran and Kirkham, 2018).

However, frequency and transitional probability effects are pervasive across sequences of all lengths. Indeed, recent studies have shown that the looking times of children are sensitive to the frequency of word trigrams as early as 12-months old (Skarabela et al., 2021). Moreover, the segmentation of these sequences plays a role in learning: children's production of irregular plurals is facilitated when the prompt is predictive of the specific word (Arnon and Clark, 2011), and words that appear in highly predictable sequences are uttered earlier (Grimm et al., 2017). Thus, statistical and probabilistic learning is not limited to segmentation and extraction of individual words but includes multiword sequences and the probabilistic relationships between the parts they have segmented.

The importance of multiunit sequences in early acquisition even shows up in adult processing, where early acquired multiword phrases are processed faster than later acquired ones—even after controlling for how frequent both occur in adult speech (Arnon et al., 2017). Exposure to multiword sequences rather than isolated words also facilitates adult learning of grammatical gender (Arnon and Ramscar, 2012) and pronouns (Myles et al., 1998). Thus, statistical segmentation of the experienced utterances will not necessarily yield a collection of words at first, but one of sequences of different lengths, including multiunit expressions. Indeed, the emphasis in second-language (L2) teaching on acquiring single words and the ways to combine them may help explain part of the difficulty that many L2 learners face (Arnon and Christiansen, 2017).

This process of extraction can then be complemented by one that combines them into longer utterances. The exemplar-based model presented by Bod (2009) follows such a philosophy, forming sentences by combining parsed fragments of utterances. However, its reliance on syntactic trees assumes a large part of the challenge that learners face, that is, forming the inventory and discovering the relationships between their components. In this sense, a better model of the process being discussed is McCauley and Christiansen (2015, 2017, 2019a) Chunk-Based Learner (CBL), which is based around extracting and combining sequences of different lengths. This computational model tracks the backwards transitional probabilities between words in adult speech directed at children and stores as “chunks” those word combinations where the transitional probability between words is higher than average. The model also can generalize to longer unseen chunks when it has previously come across their subcomponents. The transitional probabilities between chunks are used to simulate the production of novel sentences by the child, achieving high performance across 29 typologically diverse languages (McCauley and Christiansen, 2019a). This suggests that Clark's (1977) two basic operations—extraction from and combination of multiword expressions—can account for a large proportion of the speech of children during all alleged phases of analyticity.

However, combining already familiar sequences can only get a learner so far. Productivity—although not nearly as boundless as traditional approaches to language assume—is still a phenomenon to be explained: how can children generalize “I don't know that” into “I don't get that” instead of “I don't know get” without utilizing a lexicon and a grammar? A plausible proposal emphasizes the role of two interdependent processes: familiarity and generalization. As discussed before, frequency is used as a proxy for familiarity and entrenchment of a sequence in an average learner. Bybee (2006) expands this notion by arguing that familiarity with sequences can take two different forms. On the one hand, *token frequency* measures a language user's experience with a specific sequence, such as “I don't know.” This kind of familiarity influences the formulaicity of a sequence, as its internal components may get partially blurred and their phonology reduced (Bybee and Torres Cacoullos, 2009). Token frequency is the measure used to select stimuli in most of the studies reviewed in the previous section. On the other hand, *type frequency* is a more abstract feature of overlapping sequences. For example, the sequences “I have a car,” “I have a sister,” and “I have a degree,” are of the same type: the schematic sequence “I have a X” (Bybee and Thompson, 1997). These patterns are often called “frames,” with the variable components treated as “slots” to be filled with material segmented from different sequences (Bannard and Lieven, 2012; Ellis, 2012; Diessel, 2015). They can be further generalized, such as the sequences “I have a X,” “I get a X,” “I give a X” abstracting into “I Y a X” (Bybee, 2006; Divjak and Caldwell-Harris, 2015).

The first step of this process of generalization is learning one specific token of the type—say, for example, the token “want cookie” of the type “want X” (MacWhinney, 2014). As predicted by the performance of CBL (McCauley and Christiansen, 2019a), a large proportion of the productions of children are reuses of previous utterances of their caretakers (Lieven et al., 2003). Furthermore, the use of these frames by caretakers are themselves highly skewed toward specific tokens of these types (Cameron-Faulkner et al., 2003; Goldberg, 2006; Ellis and Ogden, 2017). This pattern has been independently shown to facilitate learning of phrasal patterns (Casenhiser and Goldberg, 2005). Once established, the overlap with other tokens of the frame could be used to establish which parts of the frames are “slots” and which are “components” that can be then inserted into the slots (Dabrowska and Lieven, 2005; Onnis et al., 2008).

A crucial aspect of this proposal is that the extracted chunks and abstracted slots are not necessarily identical to their characterization in a more formal grammar of a language (Bybee, 2010). Children fill slots with items organized around the most frequent token (Lieven et al., 1997), which is evidence against these slots having properties in terms of abstract categories like “verb” or “noun.” Instead, the segmented components are organized around functional (e.g., “things that are held,” MacWhinney, 2014) and semantic (e.g., “person/object,” Lieven et al., 2003; Dabrowska and Lieven, 2005) relationships in addition to distributional patterns. These components can, but need not, correspond to individual words, as children are sensitive to the statistical properties of multiword sequences (Bannard and Matthews, 2008; Skarabela et al., 2021) in addition to associative (Wojcik and Saffran, 2013) and positional (Wojcik and Saffran, 2015) information. Thus, apart from lexical items segmented from longer sequences, the components can include words along with their article (in Spanish, “la-pelota,” as opposed to “la” and “pelota,” Arnon and Ramscar, 2012) and longer chunks spanning multiple words, accounting for the jump from e.g., “I want milk” to “more milk” to, finally, “I want more milk”^3^ (MacWhinney, 2014). The developmental trajectory of the acquisition of sentence building is based on these patterns getting more abstract and connections between them arising, mirroring “the argument structures of traditional grammatical description” (Bannard and Lieven, 2012). However, the promiscuity of the “chunkatory” does not disappear in adults: the effects of multiword expression frequency and predictability in adults reviewed above suggest that segmenting individual lexical items from multiword sequences does not replace the longer sequences. In addition, behavioral and neuroimaging studies (see Vigliocco et al., 2011; for a review) suggest that knowledge of lexical items is organized around semantic and functional properties, and that the use of seemingly productive patterns is driven by distributional and associative properties (see Goldberg, 2019; for a review).

A handful of computational models illustrate this process of abstraction over sequences of various lengths. The increasing abstraction and connectedness of frames with slots is the base of the ADIOS (Solan et al., 2005) model. ADIOS generalizes over the sentences of a corpus represented as a graph, looking for sets of subsequences, including words, which appear in the same frames. After identifying a set in one level, the model looks for a new path through the sentences of the graph in a further level of abstraction. This model was able to produce sentences with similar human acceptability as the ones in the training corpus (Solan et al., 2005). However, a limitation of ADIOS is that the paths it draws rely on passes through a complete corpus, which detracts from its psychological plausibility as a model of learning. A model that better meets this requirement is a modification of CBL, dubbed CBL-LF (McCauley and Christiansen, 2019b). CBL-LF identifies lexical frames such as “a little X” or “on X own” by generalizing over sets of partially overlapping chunks. These slots could then be filled by the other stored chunks to match the sentences in the task. This modification significantly improves the performance of CBL in all the tested languages.

In summary, frames with slots obtained from the bottom-up generalization of sequences can explain productivity and acquisition without assuming a distinction between lexicon and grammar. Instead, the only assumption is a repertoire of sequences of different lengths and abstraction, the product of both segmentation and generalization. Importantly, this proposal not only accommodates the phenomena around multiunit expressions but places them at its core: the key to acquisition is the differential entrenchment of sequences and the interaction between their whole-phrase and component features.

### Processing

Traditional accounts based on a lexicon and a grammar conceive of language as involving two separate types of processes: lexical and syntactic (see e.g., Fodor, 1995). Syntactic processes result in tree-like structures where individual lexical items are only represented as dummy terminal symbols such as “Noun,” Auxiliary,” or “Verb” (Chomsky, 1970; see Kimball, 1973; for an example) based on the user's knowledge of the grammar. Separately, lexical processes match the words in the sentence with their counterparts in the lexicon. The products of both are then combined in the process of “lexical insertion” (Jackendoff, 1997); in one influential approach, this is equated to a “thematic processor” (Rayner et al., 1983) that accesses the meaning of the words stored in the lexicon and integrates with the structure (for a review, see Van Gompel and Pickering, 2007).

Although not completely separated from each other, the distinction between lexical processes and syntactic processes is present in the influential Bock/Levelt account of production (Bock and Levelt, 1994; Levelt, 1998). In it, lexical processes generate an unordered collection of words along with its thematic and syntactic roles such as “agent” and “noun” whereas the structural processes produce a tree-like structure with terminal items such as “Verb stem” into which each word is inserted incrementally (Bock and Levelt, 1994). As mentioned above, Ullman (2001) Declarative/Procedural account also implements this distinction: lexical and syntactic processes even have different neural substrates, the former in the temporal regions and the latter in the left-frontal and basal ganglia structures (Ullman, 2001, p. 39).

The starting point of an alternative to this view of processing is the kind of knowledge that results from the acquisition process outlined in the previous section. This is because, from the viewpoint of usage-based approaches to language, linguistic knowledge is the “cognitive organization of one's experience with language” (Bybee, 2006, p. 711). As such, knowledge of language is intimately tied to how its use: knowing a language is knowing how to process it (Chater and Christiansen, 2018).

Construction Grammar (e.g., Goldberg, 2006), which focuses on abstractions similar to the lexical frames discussed in the previous section, is one such single-system account of linguistic knowledge. The core assumption of this perspective is that linguistic knowledge is organized around *constructions*, which are pairings of linguistic forms and meanings. What differentiates constructions from a lexicon, however, is that constructions can be of any length insofar as they are sufficiently entrenched (Goldberg, 2003). This includes morphemes, words, idioms, phrases, and phrasal patterns such as the passive and the ditransitive. Importantly, constructions are not necessarily completely specified, even though they have a meaning; instead, they allow for any number of slots, themselves linked by semantic and functional relationships. In a classic example, the construction “The Xer, the Yer” (e.g., “the bigger, the better”) has two slots for words of the set “properties,” and the pattern itself has the meaning of linked variables (Goldberg, 2003).

Knowledge of constructions is organized hierarchically, such that a phrase (“the more you practice, the better you get at the game”) is an instance of a specific phrasal pattern (“The Xer, the Yer”), which is itself an instance of a more abstract pattern that subsumes various other constructions (Sag, 2010). Moreover, constructions can be combined provided that there are no conflicts, to the point that constructions can be parts of other constructions. For example, the “Intransitive motion” construction, “Kim ran,” can be generalized as a part of the “Caused motion” construction, “Kim ran Pat off the street” (Boas, 2013). Instances and parts are related to each other through hierarchically organized networks in which the properties of the more abstract and simpler constructions are inherited by more concrete and compounded constructions (Goldberg and Suttle, 2010).

However, the traditional presentation of Construction Grammar (Goldberg, 2006) must be modified in light of the previously discussed evidence. Mainly, the assumption that only high-frequency predictable sequences are stored (Goldberg, 2006, p. 73) is not consistent with the familiarity effects observed across the whole spectrum of frequency and the difficulty of drawing a sharp boundary between idiomatic, formulaic, and novel expressions^4^. Instead, these effects suggest that knowledge about specific sequences coexists with the generalizations made over them (Abbot-Smith and Tomasello, 2006). In other words, this means that language users must be familiar with exemplars they have experienced in addition to the abstractions they have made based on those exemplars.

The crucial point of this scheme, however, is that exemplars and generalizations are all inherently meaningful. Indeed, Jolsvai et al. (2020) found that meaningfulness was more important than frequency in the processing of multiword sequences—even in the case of phrasal fragments such as “know it gets” and “without the primary.” Thus, language processing can be largely regarded as a mechanism of pattern matching and categorization: this would be enough for a language user to identify the meaning of an utterance. For example, MacWhinney and Bates' Competition Model for processing (Bates and MacWhinney, 1987; MacWhinney, 1987), in which the identification of lexical material is a result of the competition between each partially matching lexical item, can be extended to the recognition of constructions (MacWhinney, 2014) and multiunit expressions in general (e.g., “Chunk-and-Pass processing,” Christiansen and Chater, 2016). Thus, when encountering a sentence, it is taken in sequentially and its components will activate different constructions with which it partially matches. If a filled construction (i.e., a complete sentence) is matched, then the meaning of the sentence is the meaning associated with that construction. If instead, it is a partially filled one, then the components in slots are themselves matched with other constructions in a parallel but sequential stream (see Frank et al., 2012 for a preliminary model). And, as the identified constructions are themselves meaningful, there is no need for an additional step.

Language processing, then, can be seen as a process predominantly driven by sequence matching and pattern identification. In that sense, this view is compatible with constraint-based processing approaches (for a review, see MacDonald and Seidenberg, 2006) that incorporate probabilistic cues, including, importantly, the frequency and predictability of the sentence and its components. This would in turn explain the effect of familiarity on multiunit expressions, including those in the idiomatic and formulaic range. Even more broadly, this account makes language knowledge and processing dependent on domain-general mechanisms such as categorization. For example, understanding a passive sentence would involve categorizing a sequence as an instance of the “passive construction” formed and reinforced by our previous experiences with passive sentences (for the relationship between language processing and categorization, see Lakoff, 1987; Langacker, 1987, 2008). Understanding a novel sentence would then be a case of category extension, affected by well-attested phenomena such as prototypicality (Rosch, 1988; Taylor, 2015) and exemplar coverage (Perry et al., 2010; Goldberg, 2016). In this sense, that language users can understand and produce an infinite number of sentences is no more mysterious than is our ability to categorize an infinite set of experiences.

An open issue in the literature pertains to exactly how this kind of language ability might be implemented as part of our cognitive system. More specifically, the issue regarding what is stored in a proficient user's memory has resisted clear answers. As was mentioned before, the more traditional assumption that only highly frequent regular sequences are stored (Goldberg, 2006) is not consistent with the pervasiveness of familiarity effects with multiunit expressions. The alternative to this is the claim that exemplars of all kinds of sequences are stored (Abbot-Smith and Tomasello, 2006; Bybee, 2006). However, the extent and range of stored abstractions are not clear in these accounts.

In fact, recently, Ambridge (2020a) put forward an approach based entirely around exemplars and analogies (see also Chandler, 2017) with no storage of abstractions of any kind. However, the ensuing discussion (see Ambridge, 2020b) showed the deep difficulties with such a view; namely, abstraction is necessary for any kind of psychologically realistic storage of linguistic experiences. For example, identifying that a person is uttering the sentence “hello there” regardless of the idiosyncratic variations of their intonation, timbre, and accent is already an exercise of abstraction. On the opposite end of the spectrum, recent proposals (e.g., Baayen et al., 2013) have argued that storage can be eschewed in favor of direct manipulation of the connections between sequential inputs and outcomes, embodied, for example, in Naïve Discriminant Learning (Arnon and Ramscar, 2012; Baayen and Ramscar, 2019). However, the effectiveness of this approach has yet to be shown in more expansive tasks (see Christiansen and Arnon, 2017; for a similar point). A final alternative is conceiving of sequential processing as navigating between stored experiences of sequences of any length, with the connection between sequences determined by sharing their context of occurrence. In such a scheme, lexical frames and abstractions are not descriptions of stored elements but of computational procedures and the organization of linguistic experience (Christiansen and Chater, 2016; Ambridge, 2020b; Lieven et al., 2020; McClelland, 2020).

Although our focus has been on psychological theories of language processing and learning, there are related efforts in the field of linguistics that complement these alternatives. One of them has been put forward by Jackendoff and Audring (2020, see also Culicover et al., 2017), extending the Parallel Architecture (Jackendoff, 1997) and Simpler Syntax (Culicover and Jackendoff, 2005) approaches to deal with sequences both shorter and longer than words. This proposal eschews the distinction between lexicon and grammar by including rule-like productive behavior in the lexicon alongside individual lexical items, morphemes, formulaic expressions, and high-frequency regular sentences. Schemas are formed by association over linguistic items, and some of them—“productive” schemas (Jackendoff and Audring, 2020; Chapter 2)—have productive behavior due to having completely open variables.

This theory, however, is not fully compatible with the evidence reviewed in this article. As shown above, dual-system (or, in this case, dual-process) solutions are not stable, as there is no sharp boundary to be found distinguishing the processing of higher frequency or predictability sequences that could point to the frequency threshold for storage posited by these proposals (cf. Jackendoff and Audring, 2020, p. 82). Even putatively fully productive schemas, such as object- and subject-relative clauses are influenced by the degree of entrenchment of its tokens (Reali and Christiansen, 2007a,b), which blurs the distinction between productive and non-productive patterns. Given this, the main theoretical reason for postulating a more abstract, fully productive level of language processing would be to account for the hypothetical infinite productivity of linguistic knowledge and a further distinction between performance and competence (e.g., Pylyshyn, 1973). On the former, we take the evidence for the ubiquity of multiword expressions to be an important caveat for assuming that infinite productivity is a property of human language. The latter distinction is outside of the scope of this article; however, as alluded to above, we believe that there are enough arguments to abandon it as a guiding methodological principle for the study of language (see e.g., Bybee, 2006; Chater and Christiansen, 2018).

Instead, we take Usage-based Construction Grammar (Diessel, 2015) to provide a better linguistic counterpart to our proposal. For example, Goldberg's (2019) most recent proposal conceives of constructions as patterns abstracted from clusters of tokens of sequences with overlapping memory traces. Productivity in this proposal is a continuum that depends on properties of the construction cluster such as coverage, uniformity, and frequency (Goldberg, 2019, p. 61). This is also compatible with Bybee's (2010) exemplar-based theory, where constructions are generalized emergent patterns from partially overlapping sequences that are used productively through analogy. Importantly, these patterns are stabilized diachronically in historical processes of grammaticalization (Beckner et al., 2009). Neither of these proposals assumes a sharp boundary between sequences that are stored and those that are not nor between fully productive and not- (or semi-) productive patterns, and thus are wholly compatible with the evidence presented in the previous sections.

## Conclusion

We started this article by suggesting that the distinction between lexicon and grammar has traditionally played a central role in explaining how language is understood. It provides one view of what it is to know language and how we can use it to comprehend an infinite number of sentences. Yet, as idioms and formulaic expressions show, a large portion of language is restricted to a relatively small region of infinity. Not all sentences are equal, as language users are familiar to varying degrees with sequences along the whole spectrum of frequency, predictability, and abstractness. And without a sharp boundary between what is created and what is memorized, the core explanatory scheme of the traditional view cannot hold.

Instead, we have offered an alternative perspective on the nature of language, as well as its acquisition and processing, which puts meaningful sequences of all lengths at its core. Acquiring language involves becoming increasingly familiar with the sequences used by a linguistic community, along with an increasing mastery of the ways they can be processed, organized, and combined (Chater and Christiansen, 2018). Knowing a language is learning how to use that densely interconnected network of constructions, their categories and subcategories, and their exemplars. Processing language consists in identifying and categorizing the combinations of constructions that make up a sequence. In short, multiunit expressions blur the sharp distinctions between accessing individual words or morphemes, on the one hand, and combining them into longer sequences, on the other. And they do so to such an extent that a radical reimagining of the core concepts of language is necessary.

The resulting picture is, of course, not without its difficulties. For starters, the semantics of constructions is unclear, even though their meaning is central to the account. Most examples of constructional meaning are hand-coded, with only very recent, exploratory forays into computational modeling (Perek, 2016; Rambelli et al., 2019; Busso et al., 2020) and linkages to embodied theories of meaning (Bergen and Chang, 2013). Providing an account of what constructions at different levels of abstraction mean, and how that meaning can be acquired through linguistic experience, is a crucial step for making this program viable and coherent with the assumptions of usage-based approaches.

Furthermore, as was mentioned above, the challenge of specifying the kind of cognitive architecture that implements these processes is still open. The limited but notorious success of eliminativist models (Baayen et al., 2013), in addition to the explanatory force of exemplars (Ambridge, 2020a), highlight the difficulty of establishing the limits of what is learned and how. Advancing on this issue will allow us to research other pressing issues on the implementation of this program, such as how much exposure is needed for an exemplar to be used in processing (e.g., Is a single exposure enough for matching?) or how abstract these representations can become (e.g., How many levels of abstraction are needed to account for language use? Is there a completely abstract sequence?).

Apart from the identification and matching of sequences, there are other proposals that characterize the computations behind language processing that are compatible with this proposal. One example is the recent work by Fedorenko et al. (2020) which suggests that the computations of the language network in the brain is guided by the semantic features of the words in a sequence rather than by their syntactic structure. In this proposal, “semantic composition” (Mollica et al., 2020), combining the meaning of the words in a sentence without strict syntactic parsing, is the core computation of the language network.

Another candidate is predictive sentence processing (Shain et al., 2020). But a problematic aspect of this proposals is that, traditionally, they posit massively parallel syntactic parsing (e.g., Van Schijndel et al., 2013) that violate the Now-or-Never bottleneck of language processing (Christiansen and Chater, 2016) and use a words-and-rules approach (Probabilistic Context-Free Grammars, Hale, 2001; Levy, 2008) to model predictions. However, other recent work suggests an alternative in item- and pattern-based prediction. For example, Schrimpf et al. (2021) found that language models trained to predict the next lexical item in a sequence have an almost perfect fit to neural activity during sentence processing. Crucially, the best performance is achieved by transformer models such as GPT-2 (Radford et al., 2019) which does not use rules and words, but instead relies exclusively on the transitional probabilities between lexical items and can be characterized as storing exemplar information of the trained sequences and performing shallow abstractions over them to extract patterns (Ambridge, 2020b; McClelland, 2020) that are then used in predictions. Intriguingly, whereas the state-of-the-art transformer model, GPT-3 (Brown et al., 2020) can be fooled into producing sentences that are factually incorrect or semantically odd, it almost exclusively produces grammatically correct sentences. More work needs to be done on conjoining these two possible mechanisms, and other candidates, with the evidence for the ubiquity of familiarity with multiword expressions to go beyond mere compatibility and develop a more fully rounded proposal of processing and acquisition that eschews the assumption stemming from theories based on words and rules.

Nevertheless, even with all these difficulties, the perspective on language that emerges inspired by the prevalence of multiword expressions is a promising avenue of research that eschews the core assumptions of the traditional bipartite viewpoint of the words-and-rules approach. Instead, it offers a view of language that is rooted in our general cognitive capacities and a developmentally plausible account of how linguistic knowledge can be acquired and perfected. And, even more importantly, it highlights the actual patterns of language use instead of an imagined, but never realized, idealized capacity for language.

## Author Contributions

PC and MC designed the article together. PC wrote the first draft, which was further edited and revised by MC and PC. All authors contributed to the article and approved the submitted version.

## Funding

This research was supported in part by a New Frontiers Grant from the College of Arts and Sciences at Cornell University awarded to MC.

## Conflict of Interest

The authors declare that the research was conducted in the absence of any commercial or financial relationships that could be construed as a potential conflict of interest.

## Publisher's Note

All claims expressed in this article are solely those of the authors and do not necessarily represent those of their affiliated organizations, or those of the publisher, the editors and the reviewers. Any product that may be evaluated in this article, or claim that may be made by its manufacturer, is not guaranteed or endorsed by the publisher.

## Footnotes

^1^See Jackendoff (1999) for a more nuanced approach to the connection between this perspective and language use.

^2^We thank an anonymous reviewer for this example.

^3^We thank an anonymous reviewer for this suggestion.

^4^See Goldberg (2019), for a more recent presentation of this approach that does not assume a frequency threshold.
